# Supplementary material for: Legume Consumption and Blood Pressure Control in Individuals with Type 2 Diabetes and Hypertension: Cross-Sectional Findings from the TOSCA.IT Study
Source: Nutrients. 2023 Jun 26;15(13):2895. doi: 10.3390/nu15132895 (PMC10343530; doi:10.3390/nu15132895)
Supplement: Supplementary file 1 [file nutrients-15-02895-s001.zip › nutrients-2456598-supplementary.pdf]

Table S1. General characteristics of the study participants (*N* = 1897)

Men (%)	1079 (56.9%)
Age (years)	62.8±6.4
Diabetes Duration (years)	8.7±5.7
History of Hypertension (years)	7.2±6.4
BMI (Kg/m^2)	30.8±4.5
Waist circumference (cm)	105.6±11.1
Systolic blood pressure (mmHg)	135.5±15.0
Diastolic blood pressure (mmHg)	80.1±8.8
HbA1c (%)	7.68±0.51
Total cholesterol (mg/dL)	178.7±37.4
LDL-cholesterol (mg/dL)	101.8±31.6
Triglycerides (mg/dL)	153.5±75.2
HDL-cholesterol (mg/dL)	45.8±11.7
C-reactive protein (mg/dL)	0.42±1.94
Current smokers (%)	302 (15.9%)

Table S2. General characteristics and metabolic profile of the study participants according to the sex-specific quartiles of legume intake (g/1000 kcal /day).

	**Quartile 1 (2.9±1.7)**	**Quartile 2 (8.1±1.8)**	**Quartile 3 (14.1±2.7)**	**Quartile 4 (28.3±10.6)**	**P-value**
Age (years)	63.0±6.4	62.7±6.5	62.9±6.4	62.7±6.3	0.899
Diabetes Duration (years)	8.7±5.7	8.4±5.8	8.7±5.6	9.0±5.6	0.509
BMI (Kg/m^2)	30.7±4.3	30.8±4.5	30.9±4.6	31.0±4.4	0.836
Waist circumference (cm)	106.0±11.4	105.9±11.1	105.2±11.3	105.3±10.5	0.534
HbA1c (%)	7.71±0.53	7.64±0.50	7.68±0.51	7.68±0.50	0.205
Total cholesterol (mg/dL)	177.8±39.4	180.6±36.6	179.5±38.6	177.1±35.0	0.442
LDL-cholesterol (mg/dL)	100.7±33.2	102.4±30.5	103.2±31.6	100.8±31.3	0.554
Triglycerides (mg/dL)	156.7±78.9	153.6±77.4	151.1±73.4	152.7±70.7	0.713
HDL-cholesterol (mg/dL)	45.4±11.5	46.7±12.3	45.3±11.4	45.6±11.5	0.214
C-reactive protein (mg/dL)	0.47±2.66	0.35±0.57	0.44±2.57	0.40±1.10	0.803
% on antihypertensive drugs	448 (94.3)	472 (95.9)	442 (96.3)	453 (96.2)	0.404
% with microalbuminuria	101 (21.3)	106 (22.4)	98 (20.7)	102 (21.5)	0.798

M±SD
